# Supplementary material for: Genomic signatures of local adaptation in recent invasive Aedes aegypti populations in California
Source: BMC Genomics. 2023 Jun 10;24:311. doi: 10.1186/s12864-023-09402-5 (PMC10257851; doi:10.1186/s12864-023-09402-5)
Supplement: Supplementary file 1 [file 12864_2023_9402_MOESM4_ESM.docx]

Supplementary Figure 1. Cross validation error plot showing the selection of K = 3 is the most well-presented population structure for this dataset.
