# Supplementary material for: Genomic signatures of local adaptation in recent invasive Aedes aegypti populations in California
Source: BMC Genomics. 2023 Jun 10;24:311. doi: 10.1186/s12864-023-09402-5 (PMC10257851; doi:10.1186/s12864-023-09402-5)
Supplement: Supplementary file 2 [file 12864_2023_9402_MOESM5_ESM.docx]

Supplementary Figure 2. a. Heatmap indicating correlation among 25 topo-climate variables collected from ClimateNA. b. Principle component analysis of the topo-climate variables for 12 populations used for the association analysis, with the projection of the correlations on the first PCA plan. The full names of the topo-climate variables are available in supplementary table 2.
