# Supplementary material for: Genomic signatures of local adaptation in recent invasive Aedes aegypti populations in California
Source: BMC Genomics. 2023 Jun 10;24:311. doi: 10.1186/s12864-023-09402-5 (PMC10257851; doi:10.1186/s12864-023-09402-5)
Supplement: Supplementary file 3 [file 12864_2023_9402_MOESM6_ESM.docx]

Supplementary Figure 3. Inferred relationship among 12 populations of the Ae.aegypti represented by a. a hierarchical clustering tree derived from the matrix Ω estimated under the core model and b. a correlation plot.
